# Production of Antigen-Binding Fragment against *O*,*O*-Diethyl Organophosphorus Pesticides and Molecular Dynamics Simulations of Antibody Recognition

**DOI:** 10.3390/ijms19051381

**Published:** 2018-05-06

**Authors:** Zi-Jian Chen, Xuan Zhang, Bing-Feng Wang, Mei-Fang Rao, Hong Wang, Hong-Tao Lei, Hui Liu, Yan Zhang, Yuan-Ming Sun, Zhen-Lin Xu

**Affiliations:** 1 Guangdong Provincial Key Laboratory of Food Quality and Safety, South China Agricultural University, Guangzhou 510640, China; czj1q2w3e4r5t@stu.scau.edu.cn (Z.-J.C.); zhang_xuan_2009@163.com (X.Z.); wbfeng@scau.edu.cn (B.-F.W.); rmfrwl@163.com (M.-F.R.); gzwhongd@163.com (H.W.); gzsyming@163.com (Y.-M.S.); 2 Guangdong Institute of Product Quality Supervision and Inspection, Foshan 528300, China; liuhuiyiqiyihui@163.com (H.L.); zhany1987@163.com (Y.Z.)

**Keywords:** organophosphorus pesticides, recombinant antigen-binding fragment, molecular docking, molecular dynamics simulation

## Abstract

Immunoassay for pesticides is an emerging analytical method since it is rapid, efficient, sensitive, and inexpensive. In this study, a recombinant antigen-binding fragment (Fab) against a broad set of *O*,*O*-diethyl organophosphorus pesticides (DOPs) was produced and characterized. The κ chain and Fd fragment were amplified via PCR and inserted into the vector pComb3XSS and the soluble Fab on phagemid pComb3XSS was induced by isopropyl *β*-d-thiogalactoside in *E. coli* TOP 10F’. SDS-PAGE, Western blotting, and indirect competitive ELISA results indicated that Fab maintained the good characteristics of the parental mAb. To better understand antibody recognition, the three-dimensional (3D) model of Fab was built via homologous modeling and the interaction between Fab and DOPs was studied via molecular docking and dynamics simulations. The model clearly explained the interaction manner of Fab and DOPs, and showed that the Arg-L96 and Arg-H52 were mainly responsible for antibody binding. This work provided a foundation for further mutagenesis of Fab to improve its characteristics.

## 1. Introduction

Organophosphorus pesticides (OPs) are widely used for agricultural and landscape pest control [1]. However, it has been reported that OPs are harmful to human health though food exposure [2]. Maximum residue limits (MRLs) have been established in many countries to control the residues of OPs in agricultural products and environmental samples. Up to now, instrument methods such as gas chromatography–mass spectrometry (GC-MS/MS) [3] and high-performance liquid chromatography–mass spectrometry (HPLC-MS/MS) [4] have been applied for OP analysis. While all these instrumental analytical techniques are accurate, with adequate sensitivity and repeatability, they are costly, low-throughput, and time-consuming. Thus, these methods are inadequate for monitoring a large number of samples. Unlike chromatographic techniques, immunoassay based on the high affinity between antibody and antigen has many advantages like high sensitivity, low cost, reliability, and effectiveness. Therefore, it has emerged as an alternative to instrumental methods for field monitoring [5]. Furthermore, multi-target analyses were achieved by immunoassay based on broad-specificity antibodies, which have been applied for high-throughput screening [6]. So far, several broad-specificity enzyme-linked immunosorbent assays (ELISAs) based on traditional polyclonal antibody (pAb) or monoclonal antibody (mAb) for OPs have been developed [7,8,9,10]. We also produced an improved broad-specificity mAb for *O*,*O*-diethyl organophosphorus pesticides (DOPs) [11] and developed specific ELISAs for screening DOPs in agricultural and environmental samples [12]. However, all of the proposed antibodies exhibited non-uniform specificities towards the different OPs. For example, the antibody obtained by our group showed broad cross-reactivity (CR) from 1.7% to 16,264.4% to 14 OPs [11], which might result in a false negative or positive analysis in practice. Efforts are currently being made to broaden the specificities and enhance the uniform response to OPs.

However, significant differences in the structures of OPs made it very difficult to prepare an ideal broad-specificity antibody with high homogeneity using conventional immunization techniques. The inherent drawback of conventional antibody preparation technologies is that the antibody can barely be optimized or evolved once it has been generated. However, the development of recombinant antibodies and protein engineering technologies provided a new solution to manipulate an antibody at the molecular level. Compared to animal-derived pAb and mAb, the production process of recombinant antibodies is relatively simple with no experimental animals and hybridoma. What is more, it is easy to improve the antibody’s affinity by modifying it at the genetic level. Clark et al. [13] used a combination of structure-based computational methods, thus optimizing the binding affinity of an antibody fragment to the I-domain of the integrin VLA1 using site mutagenesis. One of the 83 recombinant mutations showed a 10-fold improvement of affinity over the wild type. Kusharyoto et al. [14] used molecular modeling to construct a three-dimensional model of the atrazine-specific antigen-binding fragment (Fab) fragment K411B, defining the specific binding pockets for hapten. By altering the residues that are responsible for the binding pockets, they obtained a triple-mutant Fab fragment, which showed a 5-fold higher affinity towards the wild type. Using protein-engineering technologies, it is possible to alter the affinity and also specificity of an antibody, which offers the potential to produce antibodies with desired properties. Among recombinant antibodies, as the performance develops [15], the popularity of Fabs is also increasing. The engineering antibody, Fab, is a heterodimer of V_H_-C_H_1 and V_L_-C_L_ linked through a disulfide bond [16], and it appears that the constant regions of Fab proteins play an important role in varying region stability [17,18]. This can form the same and stable antigen recognition sites as the parental mAb via stabilization of inter-domain interaction of the complementary determining region (CDR) in V_H_-V_L_. That may be advantageous for antigen-binding activity stability rather than scFvs, a most commonly employed recombinant antibodies [19]. Moreover, the high stability and structural integrity of Fabs are helpful for crystallization studies, which help to verify molecular simulation results. Furthermore, they are inherently stable yet capable of enormous diversity, and their characteristics are attached to effective selection systems such as phage display, which is a method that allows the target Fab protein to be selected and isolated from a panning procedure [16,20]. A combination of Fab expression and display has revolutionized the isolation and engineering of antibodies [21]. Some of the most successful applications of this technology include cancer therapy [22], poisoning treatment [23], and immunoassay [24]. However, a limiting factor on the wide use of recombinant Fab, whether in bacteria like *Escherichia coli*, fungi, or higher eukaryotes in cell culture, has been the low yield of Fabs, with on average only a few milligrams of Fab produced per liter of cell culture [25]. Even so, this study provides a solid foundation for further mutations, which can potentially result in characteristic improvements compared to the original clone.

In this study, based on previously produced hybridoma cell (12C2) that secreted mAb against DOPs, a recombinant Fab was first produced and characterized. The total RNA was extracted from the hybridoma cell and then reverse-transcribed into cDNA via RT-PCR. The κ chain (V_L_-C_L_) and Fd fragment (V_H_-C_H_1) were amplified via PCR and then cloned into the phagemid pComb3XSS. A phage-displayed Fab was constructed. Soluble-specific Fab was expressed, and then characterized via SDS-PAGE, Western blotting, and icELISA. Molecular docking and dynamics simulation were used to analyze the structure-function relationship between Fab and DOPs. This study provides a foundation for the further mutagenesis of Fab to improve its characteristics.

## 2. Results

### 2.1. Amplification of Anti-DOPs Fab Gene Fragment and Construction of the Expression Vector

**Figure 1:**
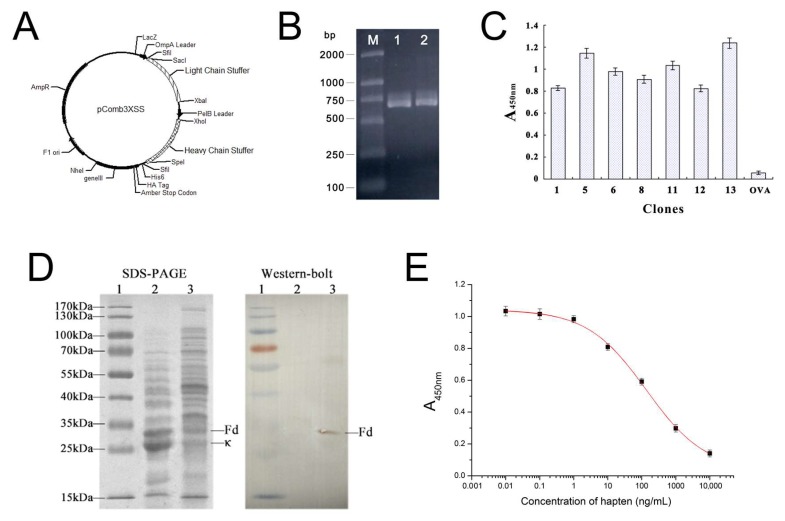
Identification and characterization of Fab. (**A**) The phage antibody expression vector pComb3XSS; (**B**) PCR amplification of anti-DOPs Fab. (M: DL2000 marker (bp); lane 1: PCR product of κ chain; lane 2: PCR product of the Fd fragment); (**C**) specificity characterization of seven positive clones with phage-ELISA for anti-DOPs Fab fragment (*n* = 3); (**D**) expression of soluble DOPs Fab via SDS-PAGE and Western blot detection, lane 1: protein marker; lane 2: total protein of *E. coli* TOP 10F’; lane 3: the DOPs-Fab fragment; (**E**) ELISA standard curve of hapten 1 based on the anti-DOPs Fab fragment (*n* = 3).

The cDNAs encoding the κ chain and Fd fragment were amplified via RT-PCR from the hybridoma cell line 12C2. The amplification of the κ chain generated an expected 650-bp fragment (Figure 1B, lane 1), while the Fd fragment generated an expected 670-bp fragment (Figure 1B, lane 2). The κ chain and Fd PCR products were sequentially cloned into the phage display vector pComb3XSS and expressed on the surface of the M13 filamentous phage.

### 2.2. Phage Displaying of Anti-DOPs Fab Fragment

The gel-purified κ chain and Fd fragment were digested, ligated into the phagemid pComb3XSS, and then transformed into *E. coli* XL1-Blue cells, as described in the Section Materials and Methods.

A total of 16 single colonies were separately cultured, and the helper phage-rescued Fab clones were tested for binding to the coating antigen in an indirect ELISA. As a result, seven positive clones were obtained without panning (Figure 1C). Hybridomas are good sources of mRNA for facile cloning of variable region genes of immunoglobulin. The correct genes could be directly cloned from hybridomas’ mRNA without further panning [26,27,28]. However, many researchers used phage biopanning or RNAse/DNAse to prevent dysfunctional genes from myeloma cells or any restricted nucleotide sequence alterations from arising during the cloning process [29]. It has previously been reported that panning could be omitted if the primer sequences were well optimized [29,30]. This could be the most promising and effective means of reducing the isolation of undesired myeloma genes and altered genes in the pool. Figure 1C shows that the displayed recombinant Fab was highly specific to the coating hapten, but not to the carrier protein OVA.

### 2.3. DNA Sequence Analysis

**Figure 2:**
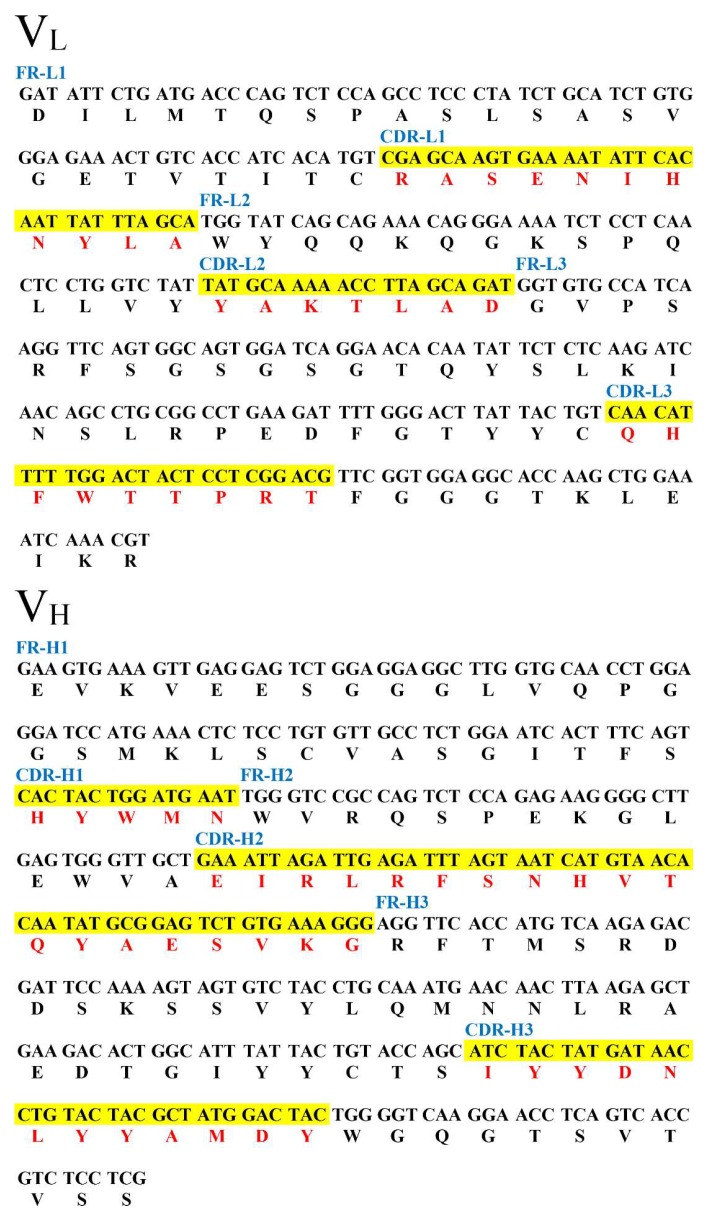
Nucleotide and deduced amino acid sequence of both VL region and V_H_ region of the Fab gene fragment for DOPs (CDRs were defined via the Kabat database). The red and highlighted words are residues (single word) and corresponding codons, respectively.

Seven positive clones were submitted for sequence analysis and the results indicated that these belong to one identical sequence. The κ chain was characterized as 650 bp, while the Fd fragment was 670 bp. These sequences were submitted to the DDBJ/GenBank/EMBL nucleotide sequence database (under the accession No. JQ692136 for the light chain and No. JQ692137 for the heavy chain). The nucleotide sequences were aligned for CDR definition and variable region numbering, using the Kabat database [31] in NCBI/IGBLAST (https://www.ncbi.nlm.nih.gov/igblast/). As shown in Figure 2, each fragment was the corresponding variable region of the Fab gene fragment for DOPs, respectively, which was determined via sequence analyses.

### 2.4. SDS-PAGE and Western Blotting Analysis of Soluble Anti-DOPs Fab Fragment

SDS-PAGE and Western blotting analysis of a recombinant protein in *E. coli* TOP 10F’ demonstrated that Fab was expressed in the soluble fraction, and obtained excellent yields. A molecular weight of 48,953 Da was determined by 12% SDS-PAGE in Figure 1D, which was very close to the theoretical values (24,991 Da for the Fd fragment and 23,962 Da for the κ chain, respectively) predicted for each fragment using the ExPASy Proteomics tools (http://web.expasy.org/protparam/).

### 2.5. Broad-Specific Binding of Fab Fragment to DOPs

The icELISA was developed to characterize the binding activity of Fab. Figure 1E displays the standard curves for hapten 1. The linear range for hapten 1 was from 7.2 ng/mL to 861.7 ng/mL. Table 1 indicated the IC_50_ and CR (%) for 14 DOPs based on both the parental mAb and Fab. Results showed that Fab maintained the basic property of the parental mAb by showing similar tends in CR.

### 2.6. Construction of the Fab 3D Structure

**Figure 3:**
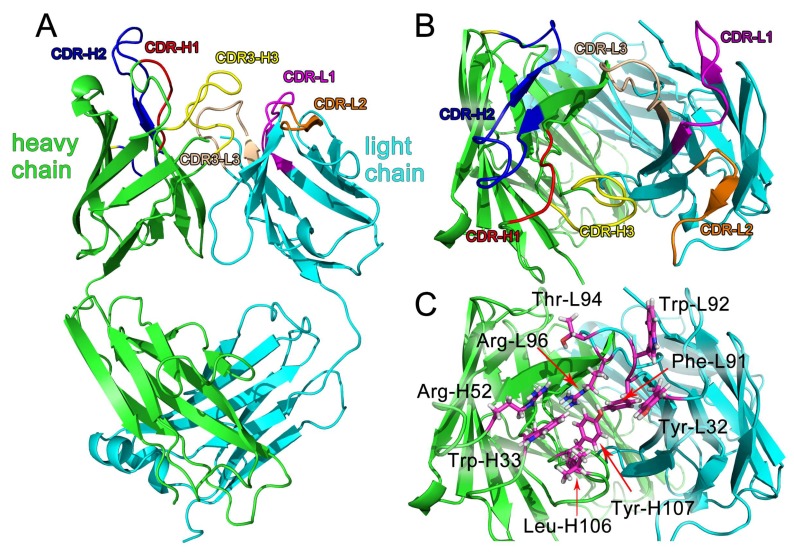
Structure of Fab. (**A**) Cartoon representation of the structure of Fab; (**B**) cartoon structure of CDRs; (**C**) main residues for DOPs binding, viewed from the antigen side, showing the arrangement of CDRs.

**Figure 4:**
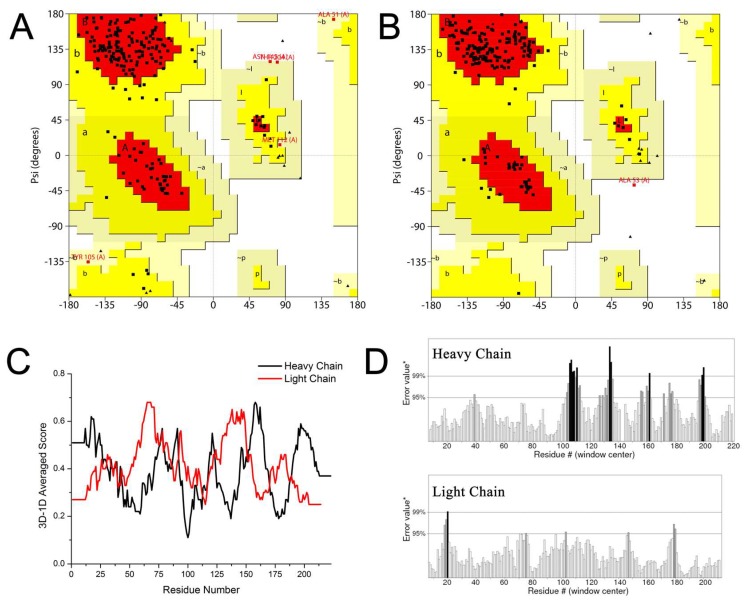
Structure verification of the homologous modeling model of Fab. The Ramachandran plot map of the heavy chain (**A**) and the light chain (**B**); (**C**) the 3D–1D averaged score of heavy chain and light chain; (**D**) results of protein structure verification of the Fab model using the ERRAT program.

The 3D homology modeling structure of Fab was built via the SWISS-MODEL [32] and is shown in Figure 3. Its application has been achieved for antibody homologous modeling [33,34,35]. As shown in Figure 3A, Fab was composed by a heavy chain (V_H_-C_H_1; Fd fragment) and a light chain (V_L_-C_L_, κ chain). A ~9.7 Å-wide and ~12.5 Å-long pocket was mainly composed of five CDRs of Fab: CDR-H1, CDR-H2, CDR-H3, CDR-L1, and CDR-L3 (Figure 3B,C). Model reliability for the heavy and light chains was assessed via PROCHECK (http://services.mbi.ucla.edu/PROCHECK/). As shown in a Ramachandran plot map (Figure 4A,B), more than 90% of the residues located in the most favored and additional allowed region. The 3D-1D scores of both the heavy chain and the light chain were mostly above 0.2 with 96.86% and 100% residues respectively, as shown in Figure 4C. Non-bonded interactions between types of atoms were computed with the ERRAT program, which showed an overall quality factor of 84.286 with the heavy chain and 96.098 with the light chain (Figure 4D). All these results indicated reasonable predicted 3D structures, hence proving their usefulness for conducting further docking analysis.

### 2.7. Interaction of Fab and DOPs

**Figure 5:**
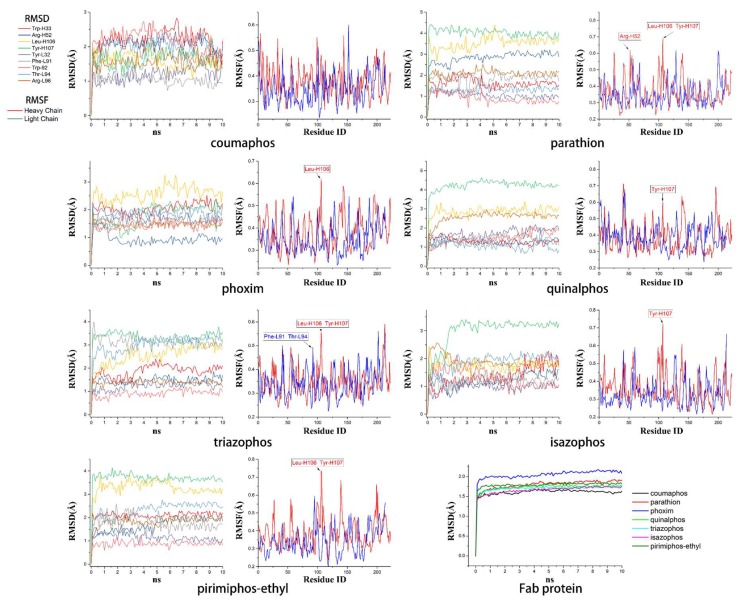
RMSD and RMSF graphs of molecular dynamics.

Computer-assisted molecular docking is an effective strategy for the study of the interaction between antibodies and small molecules. It can be used for the identification of intermolecular interactions between mAbs and target antigens [36] and provide clues for improving the binding affinity of recombinant antibodies via genetic modification [37]. During the past years, most of the reports used semi-flexible molecular docking to study the antibody-ligand interactions [38,39]. However, parameters of the ligand or protein conformation may be limited in semi-flexible docking, which may lead to insufficient information. In our study and based on the semi-flexible docking complex, we further applied molecular dynamic (MD) to construct an interaction model of Fab and DOPs. Compared to semi-flexible docking, MD provide docking aqueous solution environments and temperature for simulating conformational change during the interaction process, which would be helpful to study the antigen-Fab interaction. The equilibrium docking models calculated from MD will be analyzed for the study of interaction between Fab and DOPs. As shown in Figure 5, the values of calculated root-mean-square deviation (RMSD) graphs of main residues of CDRs and Fab protein that aliened to the initial Fab protein conformation were calculated, meanwhile, the root-mean-square fluctuation (RMSF) were also obtained from MD.

## 3. Discussion

**Figure 6:**
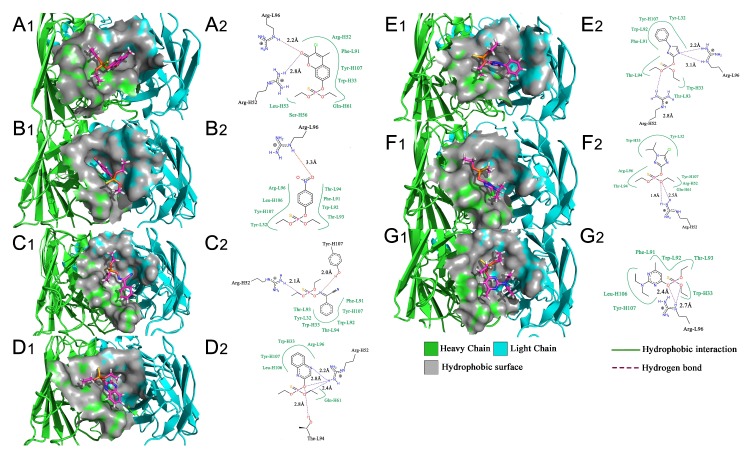
3D (subscript 1) and 2D (subscript 2) map of equilibrium docking. (**A_1_,A_2_**) Coumaphos; (**B_1_,B_2_**) Parathion; (**C_1_,C_2_**) Phoxim; (**D_1_,D_2_**) Quinalphos; (**E_1_,E_2_**) Triazophos; (**F_1_,F_2_**) Isazophos; (**G_1_,G_2_**) Pirimiphos-ethyl. The 3D and 2D images were generated via Pymol and Photoshop software, respectively.

**Figure 7:**
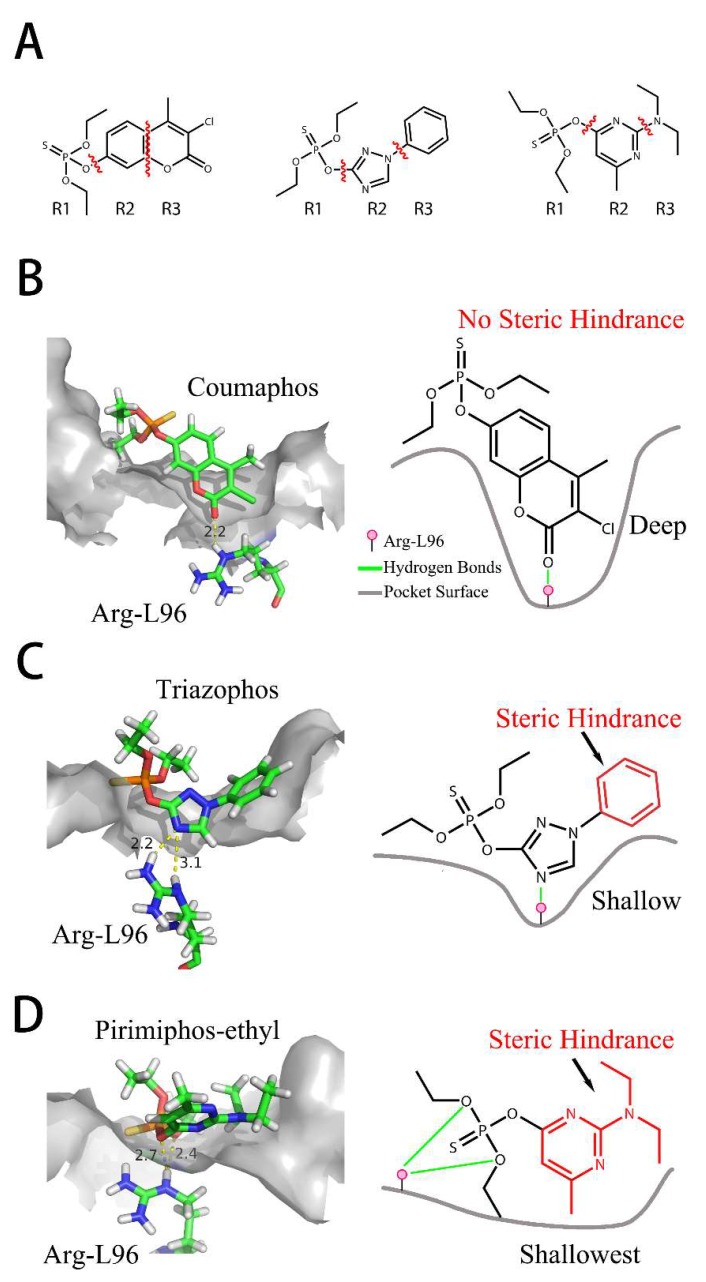
Sites of hydrogen bonds. (**A**) Split of DOPs to R1, R2, and R3 fragments in the docking analysis; (**B**) coumaphos; (**C**) tirazophos; (**D**) pirimiphos-ethyl cartoon representation and plan representation of the relationship between the sites of hydrogen bonds and their affinity.

The RMSD graphs indicated that the ligand-receptor complexes reached an equilibrium state after 10 ns of MD simulation and the equilibrium docking models are shown in Figure 6. DOPs were located on the pocket and formed π-π interactions with phenyl of Tyr-H107, Trp-H33, Tyr-L32, and Phe-H91, while a weak π–σ interaction was formed by Leu-L106. Furthermore, hydrogen bonds were formed by the *O*,*O*-diethyl thiophosphate moiety (R1, Figure 7A) with hydrophilic positive residues, Arg-H52 and Arg-L96, which were suggested to be key residues for recognizing the characteristic part of the DOPs. Unexpectedly, the docking direction of the two highest affinity DOPs (coumaphos and parathion), differed significantly from others. Due to the stronger negative charge of the oxygen atom at the terminal of R3 or R2 (Figure 7A), both of these two DOPs deeply inserted into a small cavity at the bottom of the pocket via electrostatic interaction. There, they formed hydrogen bonds with Arg-L96 by the negative group of R2 or R3, with the R1 group pointing in the opposite direction toward the outside surface of the pocket (Figure 6A_1_,B_1_). Due to the variability of CDRs, the conformation of CDR loops can be transformed via the induced-fit effect with different types of DOPs in molecular docking.

All the docking models discussed above suggested that the affinity of Fab to DOPs was related to the depth of the docking pocket, which was depended on the docking position of Arg-L96 (Figure 7B). The Arg-L96 moved towards the negative atoms of DOPs via electrostatic interaction, thus forming hydrogen bonds, while the depth of the docking pocket decreased as the Arg-L96 hydrogen position moved from R3 to R1 (Figure 7B). In the high-affinity DOPs docking models (coumaphos and parathion), strong electrostatic interaction in the bottom of the activity pocket was perceived as the main driving factor of inducing the R3 dock into the activity pocket and forming hydrogen bonds with Arg-L96, and no steric hindrance existed because there are no substituents at the phenyl ring. These OPs discussed above could easily inserted into the pocket deeply in the docking process because there are no steric hindrances exist. In the coumaphos docking model, hydrophobic interactions formed at the bottom of a ~3.2 Å depth pocket, while a stable π–π and π–σ interaction was formed between the substituent group (R2 and R3) of coumaphos and the benzene ring in the side chains of Phe-L91, Tyr-H107 and Trp-H33, which further strengthened the binding affinity of both DOPs. As in parathion docking model, the RMSF of Arg-H52, Leu-H106, Tyr-H107 was a little higher than other residues in CDRs, suggested that these residues might formed a shallower docking pocket for binding this “shorter” molecular by Arg-L96. However, the distances of the hydrogen bonds between Arg-L96 and oxygen atom of R3 were ~1.1 Å longer than coumaphos because it is too depth to insert into the bottom of the docking pocket for parathion, which might explain the higher affinity to coumaphos. In medium-affinity DOP docking models (as in phoxim, quinalphos, and triazophos docking models), Arg-L96 formed hydrogen bonds to the electronegative atoms of R2, while steric hindrance was formed by R3 (Figure 7C). The ligands were horizontally located on the shallow pocket and formed weaker π–π interaction with Tyr-H107, Trp-H33, Tyr-L32, and Phe-L91, while a weak π–σ interaction was formed by Leu-H106. Moreover, these 3D docking models and corresponding RMSD and RMSF graphs indicated that the CDRs loops where these residues were located could exhibited a flexible performance in order to expand the docking pocket for accommodating R3. In the low-affinity DOP docking models, obviously, the electronegative atoms of R2 or R3 were blocked by the substituent groups (as in pirimiphos-ethyl) and no hydrogen bonds could be formed to Arg-L96 by R2 or R3, while only R1 could formed hydrogen bonds to Arg-L96 (Figure 7D). However, due to the bigger volume of R2 and R3, the docking pocket of Fab was not “big enough” to accommodate a large volume of ligands in docking (such as isazophos diazinon and pirimiphos-ethyl). Furthermore, in comparison with other docking models, the RMSF values of Leu-H106 and Tyr-H107 were signification higher than the others, which was caused by pocket expansion in molecular dynamic process. However, the expansion of the pocket caused by the larger volume of substituted groups at benzene ring might cause a decrease in binding energy, which leads to extremely low affinity.

All the docking models discussed above indicated that the CDR-H3 exhibit high flexibility, which could be adapted to different structures of DOPs in the docking process. Moreover, the indicated stronger interaction was formed easily by a deeper docking pocket. In the MD process, electrostatic interaction is the major factor that affects the docking position with Arg-L96 and DOPs. As we summarized in the plan representation in Figure 7, the position of hydrogen bonds that Arg-L96 formed with the negative atoms of DOPs affects the distance from which the DOPs insert into the pocket. Negative atoms located at the terminal of R3 provide a suitable structure for dock into the pocket without steric hindrance, which was performed in coumaphos and parathion. In medium-affinity DOP docking models, hydrogen bonds were formed to the R2 of DOPs, generally meaning these molecules could only lie flat on a shallower pocket, which was perceived as the result of steric hindrance formed by the R3 (as in triazophos docking models in Figure 7). In the low-affinity docking models, the steric hindrance was further increased by the R2 and R3, which significantly affected the binding and recognition ability. This rule, discussed above, explains the difference of affinity performed in DOPs molecules with different structures, which was in agreement with our previous work [11].

**Figure 8:**
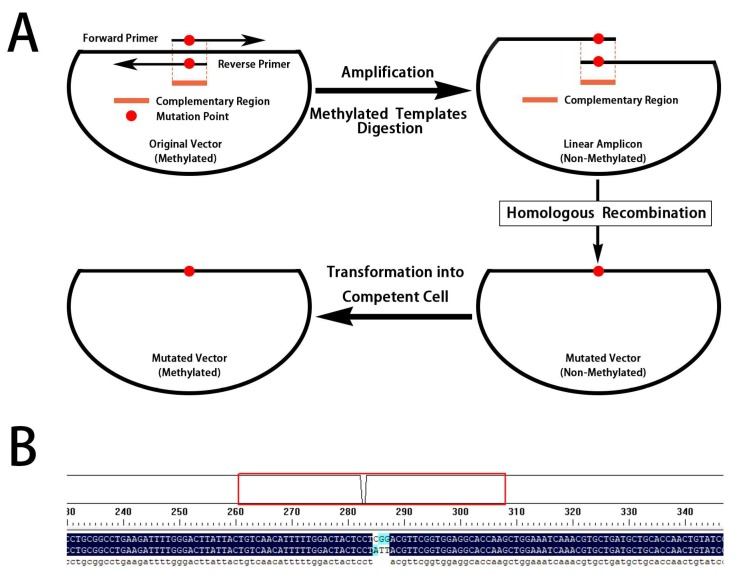
Process of site-specific mutagenesis and sequence identification. (**A**) The site-specific mutagenesis process and (**B**) the base sequence comparison before and after mutation.

Since the docking results suggest the Arg-L96 discussed above as key binding amino acid residues, we tried to mutate the Arg-L96 to lower-polarity Ile-L96 by using homologous recombination method (Figure 8A), and the forward primer and reverse primer were as follows: forward primer (5′-ACTCCTATTACGTTCGGTGGAGGCACCAAGCT-3′), reverse primer (5′-CCGAACGTAATAGGAGTAGTCCAAAAATGTTGACAGT-3′). The correct mutated plasmid sequence (Figure 8B) was transformed into Top 10F’ cell for soluble expression. In the following ciELISA test, the mutational Fab showed no binding affinity to coating antigen (Table 2). This result confirmed that the recognizability to *O*,*O*-diethyl phosphate was only performed by Arg-L96 though electrostatic interaction, which considered to be the primary recognizability to DOPs.

## 4. Materials and Methods

Analytical DOP standards were purchased from Dr. Ehrenstorfer GmbH (Augsburg, Germany). The hybridoma cell line (12C2), the coating hapten H1 (4-((diethoxyphosphorothioyl) amino) butanoic acid), and the coating antigen (hapten 2-ovalbumin) were self-prepared [11], as previously described. The XL1-Blue and Top 10F’ *Escherichia coli* strains have previously been established by our laboratory. The plasmid vector pComb3XSS (Figure 1A) was obtained from the Barbas Laboratory, TSRI, La Jolla, CA, USA. The helper phage VCSM13 was obtained from the Naval General Hospital of Beijing, China. Horseradish peroxidase (HRP), and 3,3′,5,5′-tetramethylbenzidine (TMB) were obtained from Sigma-Aldrich (Shanghai, China). Ampicillin, kanamycin, and Isopropyl β-d-thiogalactopyranoside (IPTG) were purchased from Takara (Dalian, China). DNA polymerase and DNA restriction enzyme were also purchased from Takara. HRP-conjugated goat anti-mouse IgG and anti-His tag mouse monoclonal antibodies were purchased from TransGen Biotech Co. Ltd (Beijing, China). Mut Express II Fast Mutagenesis Kit V2 for site-specific mutagenesis was obtained from Vazyme Biotech Co., Ltd. (Nanjing, China). All other chemicals were standard commercial analytical-grade reagents.

### 4.1. Construction of Anti-DOPs Fab Fragment

Total RNA from about 1 × 10^7^ hybridoma cells was extracted using TRIzol reagent and then reverse-transcribed via RT-PCR according to the manufacturer’s instructions. The PCR primers were designed as previously described [40]. PCR reactions of amplifications of κ chain and Fd fragment were performed in total volumes of 100 μL, containing 5 μL of cDNA reaction product, 0.2 μmol/L of 5′ primer and 3′ primer, respectively, 200 μmol/L of dNTPs, an optimized Mg^2+^ concentration (2–6 mmol/L), and reaction buffer supplied by manufacturers. After denaturizing at 95 °C for 5 min, 1 U of Taq DNA polymerase was added, followed by 30 cycles at 94 °C for 50 s, 55 °C for 50 s, 72 °C for 50 s, with a final extension at 72 °C for 8 min.

### 4.2. Construction of Recombinant Plasmid

The phagemid vector pComb3XSS was used to express the Fab fragment [11]. The κ chain PCR product and the vector pComb3XSS were digested with *Sac* I/*Xba* I and gel purified. Then, they were ligated by adding 1.5 U of T4 DNA ligase at 16 °C overnight to create recombinants. After ligation, 0.5 μL of DNA was transformed into 50 μL of *E. coli* XL1-Blue competent cells via standard chemical methods (CaCl_2_/heat shock). Phagemid carrying κ chain was extracted and checked for the presence of κ chain insert via *Sac* I/*Xba* I digestion. Subsequently, the phagemid vector pComb3XSS, carrying the κ chain was digested with restriction enzymes *Xho* I/*Spe* I, and ligated with a similarly digested Fd fragment PCR product. Incubation and characterization were preceded as described above. The phagemid pComb3XSS-Fab was then transformed into *E. coli* XL1-Blue competent cells. Finally, plasmids were isolated from individual clones selected on LB solid medium that contained ampicillin.

### 4.3. Phage Display

Single colonies were separately cultured in 10 mL LB medium containing ampicillin (100 μg/mL) overnight at 37 °C. Helper phage VCSM13 (10^12^ pfu) was added and the culture was shaken for an additional 2 h at 30 °C. Then, 70 μg/mL of kanamycin was added and the culture was allowed to produce Fab, displaying phage overnight with shaking at 30 °C. The supernatant was cleared via centrifugation (12,000 *g*) at 4 °C for 5 min.

To detect the phage-displaying Fabs, 100 μL of coating antigen (1 μg/mL) in 0.05 mol/L bicarbonate buffer (pH 9.6) was coated onto ELISA plates via overnight incubation at 4 °C. The well was washed twice with PBST (0.01 mol/L phosphate-buffered saline (PBS) with 0.05% Tween-20 at pH7.4) and blocked via 5% skim milk in PBST at 37 °C for 3 h. After shaking out the blocking solution, the plates were dried at 37 °C for 1 h, and then stored at 4 °C. A volume of 100 μL of phage solution (5 × 10^8^ cfu) was added to the plate and incubated at 37 °C for 1 h. The plates were washed with PBST five times, and 100 μL of 1:3000 diluted HRP/anti-M13 conjugate in PBST was added. After incubation for 1 h at 37 °C and washing five times with PBST solution, TMB solution was added to the wells (100 μL/well) and further incubated at 37 °C for 15 min. The reaction was stopped via addition of 2 mol/L H_2_SO_4_ (50 μL/well) and the absorbance was recorded at 450 nm.

### 4.4. Expression of Anti-DOPs Fab Fragment

The recombinant phagemid, which had been confirmed to contain the correct sequence (via DNA sequencing), was transformed into *E. coli* Top 10F’ competent cells subsequently for expression via soluble protein expression [25]. Colonies were grown in 20 mL of 2× YT containing ampicillin (100 mg/mL) at 37 °C until an OD_600nm_ of 0.6 was achieved. Briefly, IPTG (1 mmol/L) was added and the culture was incubated for 12 h at 18 °C in an orbital shaker incubator at 200 rpm. The cells were harvested via centrifugation. The supernatants carrying soluble Fab fraction were concentrated using an Amicon Ultra centrifugal filter device (10 kDa cutoff, Millipore, Bedford, MA, USA).

### 4.5. SDS-PAGE and Western Blot Analysis

The Fab fragment was resolved via 12% SDS-PAGE. For Western blot analysis, Fab was detected via anti-His tag mouse monoclonal antibody (1:3000), HRP-conjugated goat anti-mouse IgG (1:3000), and the blot was developed with the DAB/H_2_O_2_ system.

### 4.6. Indirect Competitive ELISA

For indirect competitive ELISA based on Fab, the coating and blocking procedure were identical to the phage-display ELISA. A series concentration of DOPs standards in PBS (0.01 mol/L, pH 7.4) containing 5% methanol was added to the plate (50 μL/well), followed by adding 50 μL/well of Fab diluted with PBST (0.01 mol/L, pH 7.4). The plate was incubated at 37 °C for 1 h and washed five times with PBST. A volume of 100 μL 1:3000 diluted anti-His tag mouse monoclonal antibody in PBST was added to the wells, which was incubated for 40 min at 37 °C and washed five times with PBST solution. Then, a volume of 100 μL 1:5000 diluted HRP-conjugated goat anti-mouse IgG in PBST was added to the wells. After incubation for 30 min at 37 °C the mixture was washed five times with PBST solution. TMB solution was added to the wells (100 μL/well) and incubated at 37 °C for 15 min. The reaction was stopped via addition of 2 mol/L H_2_SO_4_ (50 μL/well) and the absorbance was recorded at 450 nm. Competitive curves were obtained by plotting the normalized signal (A_450_) against the logarithm of the analyte concentration. The 50% inhibition value (IC_50_) and limit of detection (LOD) were obtained from a four-parameter logistic equation of the sigmoidal curves, using OriginPro 8.5 software (version 8.5, OriginLab, Northampton, MA, USA).

### 4.7. Cross-Reactivity Study

The specificity of the icELISA assays was determined using ten DOPs under indirect competitive ELISA conditions. The percent cross-reactivity (CR) values were calculated according to the following equation: IC_50_ (hapten 1, μmol/L) × 100/IC_50_ (cross reactant, μmol/L) [11].

### 4.8. Homology Modeling and Molecular Simulations

The protein sequences of both the heavy chain (Fd fragment) and the light chain (κ chain) were translated via DNAman (version 5.2.2). The structural model of the heavy chain and the light chain were built via homology modeling using the SWISS-MODEL Workspace (http://swissmodel.expasy.org/) [41] and the highest identity templates were chosen for homology modeling: heavy chain (PDB ID:1WC7, identity 90.54%, coverage 99%) and light chain (PDB ID:1WEJ, identity 96.73%, coverage 100%).

The molecular structures of DOPs (ligands) were obtained from the ZINC database (http://zinc.docking.org/). Docking simulations were performed using LeadIT 2.1.8 software (http://www.biosolveit.de/LeadIT/) via Enthalpy and Entropy (hybrid approach) ligand binding setting and the others were default. Then, the semi-flexible docking complex contained DOPs molecules and Fab antibody was analyzed via MD using NAMD2 (Nanoscale Molecular Dynamics, University of Illinois, Urbana-Champaign, IL, USA) software with the Amber ff99SB force field. The TIP3PBOX model was chosen for the solvent model along with the Oct solvent method with a minimum distance of 10 Å to the octa edge. According to our previous similar studies [42], the energy of the structure was minimized with 10,000 runs and 1 fs for 10 ns simulation time with a simulation temperature of 310 K.

### 4.9. Site-Specific Mutagenesis

Homologous recombination method was chosen for the site-specific mutagenesis of Fab. The mutational plasmid was prepared according to the Mut Express II Fast Mutagenesis Kit V2 instructions and primers were designed by CE Design V1.04 tools from Vazyme Biotech Co., Ltd. The correct mutated plasmid will perform the following bioactivity verification test. All the transformation, expression and ELISA steps were the same as discussed above.

## 5. Conclusions

In this study, the recombinant anti-DOPs Fab gene from a hybridoma cell line that secreted mAb with broad specificity against a class of DOPs, was successfully cloned and expressed. The Fab gene showed characteristics similar to the parent mAb. Molecular docking and dynamic simulations were used to construct the 3D structure of Fab and study the interaction between Fab and DOPs. Results showed that several amino acid residues such as Arg-L96, Trp-H32, Tyr-H107, Thr-H94, and Arg-H52 were mainly responsible for antibody binding. The interaction model explained the different cross-reactivity of Fab towards DOPs very well. In future studies, crystallization of Fab will be performed to verify MD and the protein structure. Moreover, based on this study, further site-directed mutagenesis can be performed to alter the binding characteristics of Fab, which might improve its application for DOP determination.

## Figures and Tables

**Table 1:** Cross-reactivity (CR) of Fab to *O*,*O*-diethyl organophosphorus pesticides (*n* = 3).

DOPs	Structure	Fab		MAb	
		IC_50_ (ng/mL)	CR (%)	IC_50_ (ng/mL)	CR (%)
Hapten 1	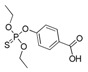	135.7	100.0	178.3	100.0
Coumaphos	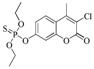	1.0	17,747.0	7.6	2931.9
Parathion	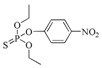	3.1	4422.1	1.1	16,264.4
Dichlofenthion	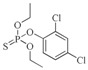	41.2	357.7	62.7	308.7
Phoxim	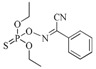	44.9	310.9	64.0	286.3
Quinalphos	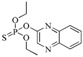	75.3	185.1	100.2	182.9
Triazophos	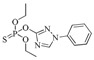	106.6	137.4	136.2	141.3
Phosalone	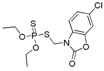	263.0	65.4	281.6	80.2
Phorate	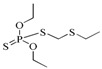	325.8	37.4	837.9	19.1
Chlorpyrifos	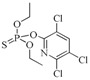	895.3	18.3	2087.6	10.3
Bromophos-ethyl	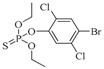	1064.8	17.3	2673.2	9.1
Sulfotep	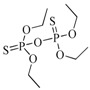	1547.8	9.7	3.5843	5.5
Isazophos	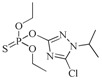	2289.5	6.4	6989.9	2.8
Diazinon	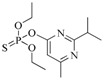	4515.6	3.2	10,865.0	1.7
Pirimiphos-ethyl	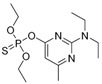	6333.0	2.5	12,266.9	1.7

**Table 2:** Comparison of bioactivity between original and mutated Fabs (*n* = 3).

Fab Dilution Multiple	Titer *	
	Original	Mutated
1	2.3045	0.1310
2	1.0645	0.1280
3	0.5510	0.1255
4	0.4265	0.1090
5	0.4280	0.1395
6	0.3145	0.1170
7	0.2860	0.1005
Blank	0.0980	0.1380

* The concentration of coating antigen was 1 μg/mL.
